# Mitochondrial dysfunction in the gastrointestinal mucosa of children with autism: A blinded case-control study

**DOI:** 10.1371/journal.pone.0186377

**Published:** 2017-10-13

**Authors:** Shannon Rose, Sirish C. Bennuri, Katherine F. Murray, Timothy Buie, Harland Winter, Richard Eugene Frye

**Affiliations:** 1 Autism Research Program, Arkansas Children’s Research Institute, Little Rock, Arkansas, United States of America; 2 Department of Pediatric Gastroenterology and Nutrition, MassGeneral Hospital for Children, Boston, Massachusetts, United States of America; 3 Department of Gastroenterology, Boston Children’s Hospital, Boston, Massachusetts, United States of America; Chiba Daigaku, JAPAN

## Abstract

Gastrointestinal (GI) symptoms are prevalent in autism spectrum disorder (ASD) but the pathophysiology is poorly understood. Imbalances in the enteric microbiome have been associated with ASD and can cause GI dysfunction potentially through disruption of mitochondrial function as microbiome metabolites modulate mitochondrial function and mitochondrial dysfunction is highly associated with GI symptoms. In this study, we compared mitochondrial function in rectal and cecum biopsies under the assumption that certain microbiome metabolites, such as butyrate and propionic acid, are more abundant in the cecum as compared to the rectum. Rectal and cecum mucosal biopsies were collected during elective diagnostic colonoscopy. Using a single-blind case-control design, complex I and IV and citrate synthase activities and complex I-V protein quantity from 10 children with ASD, 10 children with Crohn’s disease and 10 neurotypical children with nonspecific GI complaints were measured. The protein for all complexes, except complex II, in the cecum as compared to the rectum was significantly higher in ASD samples as compared to other groups. For both rectal and cecum biopsies, ASD samples demonstrated higher complex I activity, but not complex IV or citrate synthase activity, compared to other groups. Mitochondrial function in the gut mucosa from children with ASD was found to be significantly different than other groups who manifested similar GI symptomatology suggesting a unique pathophysiology for GI symptoms in children with ASD. Abnormalities localized to the cecum suggest a role for imbalances in the microbiome, potentially in the production of butyrate, in children with ASD.

## Background

Autism Spectrum Disorder (ASD) is characterized by impairments in communication and social interactions along with restrictive and repetitive behaviors [1]. In the United States, ASD is now estimated to affect almost 2% of children [2]. Although behaviorally defined, ASD is associated with several medical co-morbidities [3] such as allergies [4], epilepsy [5], gastrointestinal (GI) disorders [6], attentional problems [7] and anxiety [8] as well as physiological abnormalities of the mitochondria [9, 10], immune system [11] and redox metabolism [11, 12].

As many as 91% of children with ASD may be affected by debilitating GI symptoms such as constipation, diarrhea, or food allergy and/or intolerance [13, 14]. Developmental delays associated with ASD do not account for these symptoms, as GI symptoms are significantly more common in children with ASD as compared to children with developmental delays without ASD [15]. Many GI abnormalities reported may be unique to individuals with ASD. For example, dysfunction in enterocytes carbohydrate transportation [16], inflammation that is not fully consistent with a classic GI disorder [13, 14] and imbalances in the enteric microbiome [17–19] have all been reported.

As discussed in our recent review, the connection between GI symptoms and ASD through mitochondrial dysfunction is compelling since ASD is strongly associated with both GI symptoms and mitochondrial dysfunction, and mitochondrial dysfunction is strongly associated with GI symptoms [20]. Gut dysmotility is common not only in children with either ASD or mitochondrial disease [21, 22], but also in children with both disorders concomitantly [9, 10]. Mitochondrial dysfunction can cause inflammation, disrupt enterocyte function and cause dysmotility, resulting in many of the GI abnormalities reportedly associated with ASD. Interestingly, perturbations in the enteric microbiome can link ASD, mitochondrial dysfunction and GI abnormalities as discussed below.

**Fig 1:**
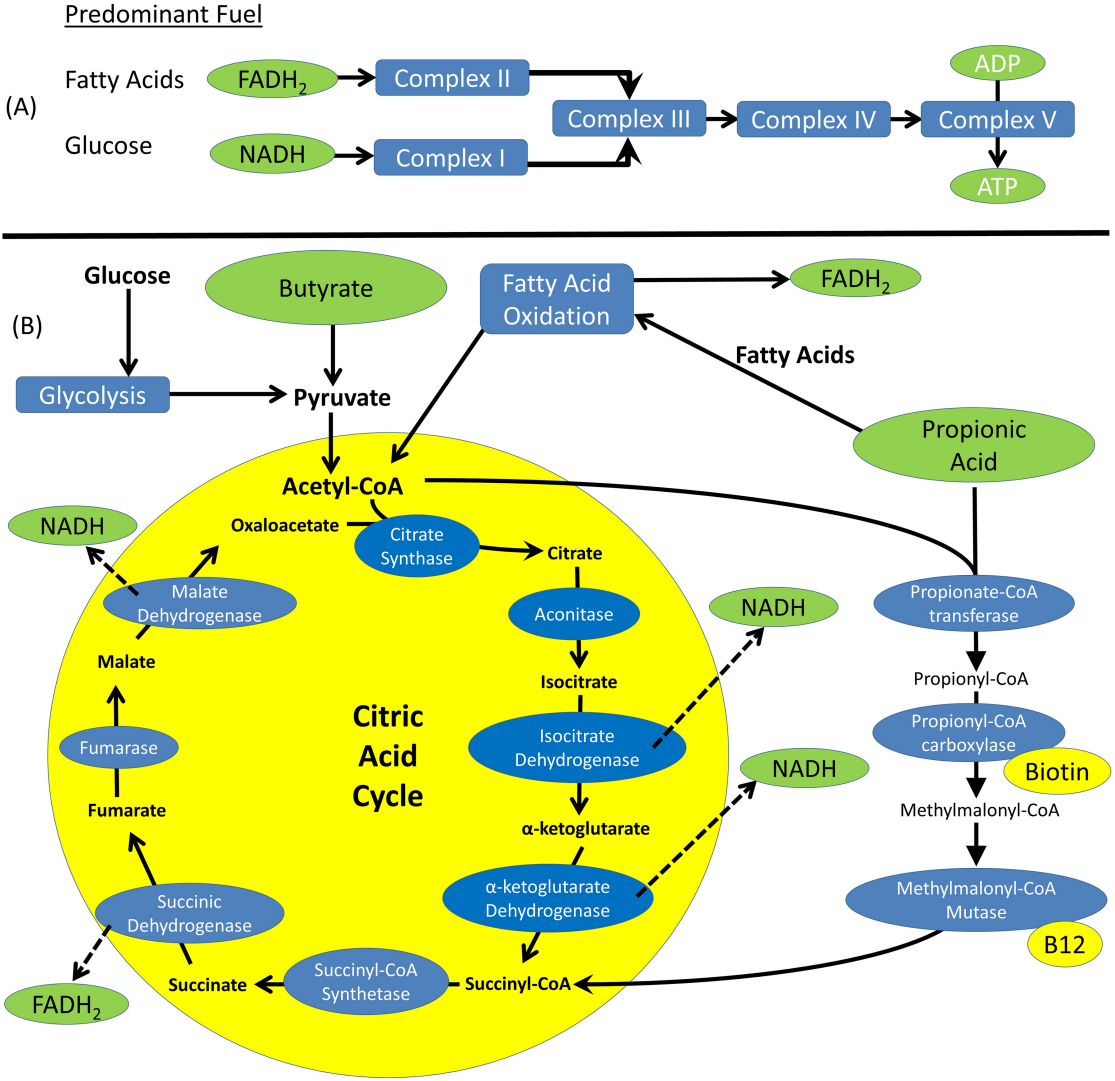
Mitochondrial pathways utilize short chain fatty acids as substrates. **(A)** The electron transport chain has two distinct starting points, Complex I and Complex II, each of which have a predominant fuel source. Complexes III, IV and IV are common to both of these pathways. (B) Butyrate and Propionic Acid enter mitochondrial metabolism through two slightly overlapping pathways. Butyrate enters the citric acid cycle through its key substrate Acetyl-CoA, similar to glucose. The citric acid cycle predominantly produces NADH that is the substrate for Complex I. Propionic acid can be metabolized through two different pathways, both of which result in a relatively greater production of FADH_2_ that is the substrate for Complex II. Propionic acid can produce fatty acids that are then the substrates for fatty acid oxidation. Propionic acid can be metabolized through several enzymes resulting in bypassing the first half of the citric acid cycle and using up Acetyl-CoA.

The cecum is a site of active microbiota fermentation resulting in the production of short chain fatty acids (SCFAs), including both propionic acid (PPA) [23, 24] and butyrate (BUT) [25]. Several studies have shown that the enteric microbiome is disrupted in children with ASD such that there is an overrepresentation of bacteria such as *Clostridia* spp which produce PPA and BUT [26–31]. Both BUT and PPA can modulate metabolism albeit in slightly different ways. For example, both can act as mitochondrial fuels, although they enter the mitochondrial energy pathways at slightly different points (Fig 1). In addition, both PPA and BUT have differential modulatory effects on mitochondrial and cellular function which can be complex and concentration dependent [20, 24] and can include modulation of T cell function [32] and cytokine production [33]. Indeed, SCFA can disrupt cellular physiology in order to cause lower GI tract symptoms associated with ASD such as non-specific inflammation and dysmotility [13, 14, 19].

**Fig 2:**
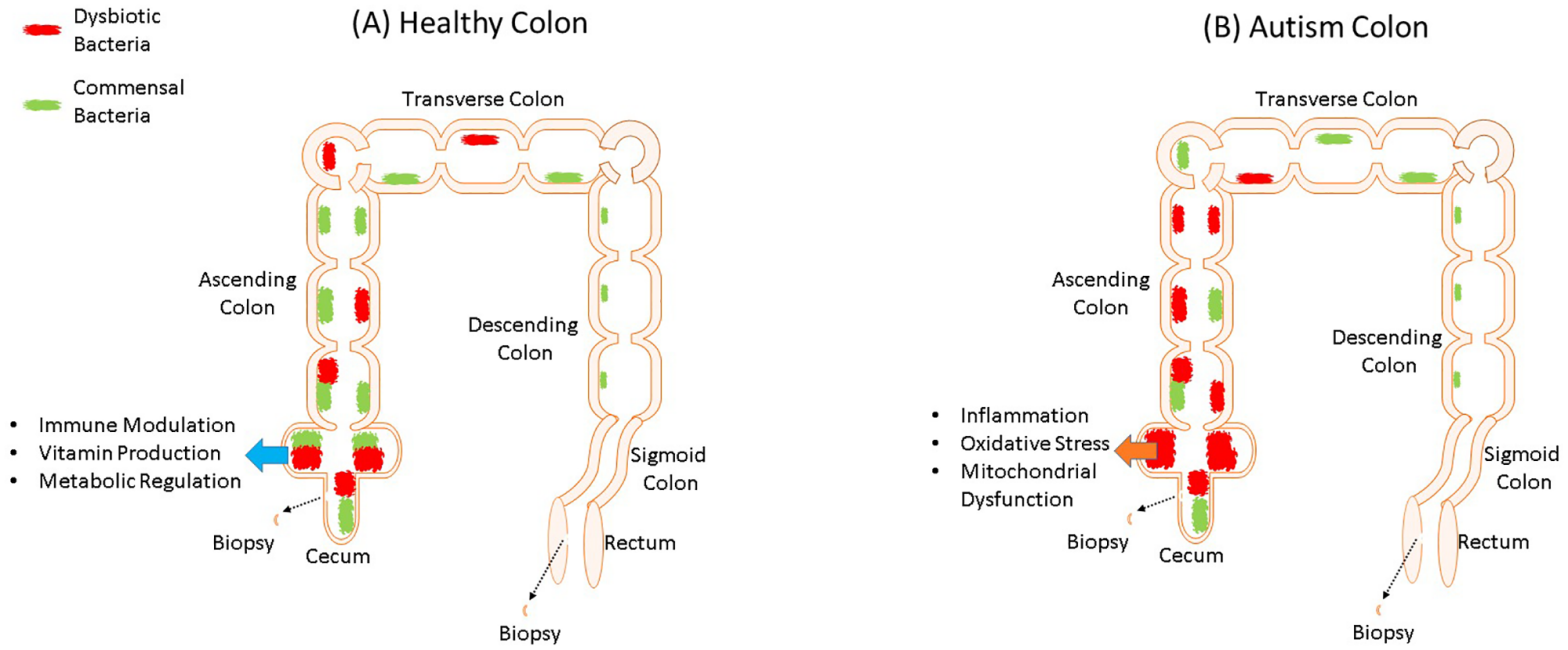
The microbiome in the lower gastrointestinal (GI) tract of healthy (A) and individuals with autism (B). (For review of difference in the microbiome between children with autism and neurotypical children please see our recent reviews [35, 36]). For both neurotypical and autistic children, we obtained biopsy samples from the cecum and rectum. It is believed that the GI tract of individuals with autism have a greater amount of dysbiotic bacteria as compared to commensal bacterial. While the microbiome of the healthy GI tract provides positive immune and metabolic regulation, the imbalance in bacteria in the GI tract of individuals with autism results in oxidative stress, inflammation and mitochondrial dysfunction. Since the cecum is one of the most metabolically active regions of the GI tract for the microbiome, we are particularly interested in measuring mitochondrial function in the cecum. We compared mitochondrial function in the cecum to the rectum since the rectum has a much less metabolically activity microbiome. To understand if mitochondrial abnormalities are unique to children with ASD, we compared measurement of mitochondrial function in children with ASD to two groups of children without ASD, those with Crohn’s disease and those with non-specific GI complaints.

To determine whether mitochondrial dysfunction may contribute to GI symptoms in children with ASD we directly measured mitochondrial function in GI mucosa of children with ASD (See Fig 2). We hypothesized that not only would we find mitochondrial dysfunction in the GI tract of children with ASD, but also that mitochondrial dysfunction would be specific to an area of the GI tract where the microbiome is most metabolically active. Specifically, since the cecum is the major site of microbiota fermentation, where the production of SCFAs such as PPA and BUT is greatest, we hypothesized that the mitochondrial function would be most disrupted in the cecum. The mucosa was studied since it is the interface between the enteric microbiome and host. To control for individual variability in mitochondrial function, we compared mitochondrial function in the cecum to mitochondrial function in the rectum, less commonly a site of GI pathology in children with ASD and an area of the GI tract where the microbiota is not metabolically active [34].

## Material and methods

The Institutional Review Board of the Massachusetts General Hospital (Boston, MA) approved the collection and storage of participant samples for later analysis. Written informed consent was obtained from parents of the participants and written assent was obtained from participants > 7 years of age. Samples were collected in the endoscopy suite, immediately snap frozen in either dry ice or liquid N_2_, and stored at -80°C in the biorepository at the Massachusetts General Hospital. Ten de-identified samples from each group were sent to Arkansas Children’s Research Institute (Little Rock, AR) for analysis. Average storage time was 2.5 year (SD 0.9 years). Assigned sample identifiers were independent of the group.

Mitochondrial function was analyzed at Arkansas Children’s Research Institute (Little Rock, AR) by measuring the activity and content of the electron transport chain (ETC) complexes, the final common pathway for energy production, as well as the activity of citrate synthase, a citric acid cycle enzyme used as an indicator of mitochondrial content. The University of Arkansas for Medical Sciences Institutional review board determined that the anonymized samples were exempt from further institutional review board review. Researchers at Arkansas Children’s Research Institute were blinded to the group allocation during preparation and measurement of mitochondrial activity assays. In order to balance the same number of samples from each group on Western blot gels, although the identity of the samples was not provided, a key was provided to indicate which samples should be grouped together. Initial statistical analyses were performed with knowledge of which samples should be grouped together but without knowledge of the identity of individual groups.

### Participants

Ten children diagnosed with Autistic Disorder with Gl complaints, 10 children with Crohn’s disease and 10 children with non-specific gastrointestinal complaints matched on age and gender underwent elective diagnostic colonoscopy. GI clinical characteristics of participants are provided in Table 1 with more detailed clinical information in S1 Table. 70% of the participants were male and average age was not significantly different across groups [Mean (SD): Autism 12.7 (4.2) years; Crohn’s 12.5 (3.8) years; Non-specific 12.8 (4.1) years]. One participant with high-functioning ASD (ASD ID 11) was assigned to the neurotypical group because of an error in coding. This was not discovered until the samples were unblinded so the individual remained in the neurotypical group for all of the analysis in order to follow an intent-to-treat model.

**Table 1:** Patient symptoms and gastrointestinal abnormalities.

Age /Gender	Gastrointestinal Symptoms	Upper Endoscopy	Upper Histology	Lower Endoscopy	Lower Histology
**Autism Patients**					
17yo M	AP, Gagging, Choking	NL	NL	NL	NL
14yo M	C, AP, GERD	NL	Chronic gastritis	NL	NL
11yo M	Severe C, Anal stenosis	Hiatal Hernia	NL	NL	NL
12yo F	C, D	NL	Mild chronic inactive gastritis	NL	NL
17yo M	Severe C	NL	Focal minimal chronic inactive gastritis	NL	NL
5yo F	C	NL	NL	NL	melanosis coli
12yo F	C, GERD, Weight loss	NL	1 EOS per hpf in distal esophagus	NL	NL
17yo M	GERD, AP	Esophagitis	NL	NL	NL
7yo M	Lower AP	NL	NL	NL	NL
15yo M	GERD, Gastritis, Chronic D	NL	NL	NL	NL
**Neurotypical with Non-Specific GI symptoms**					
7yo M	C, Rectal bleeding	Thickened folds in esophagus	Esophagitis < 20 EOS per hpf	Nodule in sigmoid; nodular ileum	NL
11yo F	Recurrent oral ulcers	Esophagitis; gastritis	NL	NL	NL
11yo M	AP; C	NL	NL	NL	NL
18yo M	AP, D	NL	NL	NL	NL
13yo M	C	NL	NL	NL	Increased cellularity in the lamina propria; non-specific
17yo M	AP, C, lactase deficient	NL	NL	NL	NL
6yo F	AP	NL	NL	NL	NL
17yo M	AP	Gastritis	Reactive gastropathy	NL	NL
13yo F	AP	Antral erythema	NL	NL	NL
15yo M^a^	C, rectal bleeding	NL	NL	NL	NL
**Neurotypical Patients with Crohn’s Disease**					
6yo M	History of AP, D, perianal fistula; but no active symptoms	Duodenitis	Focal active gastritis; mildly active duodenitis	Ileitis; colitis	Ileitis with granulomas; cecum and rectum: mildly active colitis;
12yo F	Weight loss; AP	NL	Esophagitis	Ileitis; colitis	Rectum: mildly active chronic colitis
16yo M	D, AP, perianal fistula	NL	Chronic inactive gastritis	Ileitis	Irectum: focal active colitis
14yo M	History of AP, D but no active symptoms	NL	Mild chronic gastritis	NL	Granuloma in ileum;
7yo F	Hematochezia	NL	Active duodenitis	Erythema rectum to cecum.	Focal active ileitis; cecum and rectum: mildly active colitis
11yo F	History of D, V fever & AP but no active symptoms	NL	NL	Erythema of colon.	mild active chronic colitis in rectum
17yo M	History of weight loss, D and skin tag, but no active symptoms.	Duodenitis	Mild chronic duodenitis; chronic inactive gastritis; granuloma in esophagus	Friable ileum.	Chronic ileitis with granuloma; cecum and rectum: normal
16yo M	AP, diarrhea	NL	Focal active gastritis	Anal fissure. sigmoid & transverse colon ulcerations	Ileum: granuloma;
15yo M	History of AP, D	NL	Chronic inactive gastritis	Ileum: congested and erythematous.	Active ileitis;
11yo M	No active GI symptoms	NL	Chronic inactive gastritis	NL	Ileum: granuloma; cecum and rectum chronic quiescent colitis

^a^This high-functioning child with autism was misclassified as typically developing during the blind analysis. Thus, in all analyzes this individual was included in the typically developing group.

Abbreviations: AP = Abdominal pain; C = Constipation, D = Diarrhea, EOS = Eosinophil; F = Female, GERD = Gastroesophageal reflux disease; GI = Gastrointestinal, hpf = high powered field; M = Male, NL = normal; V = Vomiting, yo = years old.

Inflammation was determined by visual endoscopy inspection as well as histological examination of biopsied tissue. Endoscopy and histological examination was conducted in multiple bowel locations and is summarized in Table 1. None of the children with autism had inflammation in the colon. One had melanosis coli consistent with laxative treatment for constipation. Lymphoid hyperplasia in the colon without inflammation was considered to be normal. Of the 10 neurotypical children without a diagnosis of inflammatory bowel disease, only one had mild, non-specific changes in the colon that could be related to dysbiosis associated with constipation. The 10 children with Crohn’s disease all had some evidence of inflammation consistent with Crohn’s disease. Nine of ten had inflammation in the ileum; whereas, in the cecum, 7 of the 10 children had a normal biopsy, and in the rectum only 4 of the 10 children had a normal biopsy.

### Clinical procedures

Specific clinical procedures for defining neuropsychiatric and regression status in this cohort have been previously described [37]. Briefly, neuropsychiatric status was established for all subjects using Diagnostic and Statistical Manual-Fourth Edition, Text Revision diagnostic criteria. Cases meeting full criteria for Autistic Disorder underwent the Autism Diagnostic Interview-Revised (ADI-R) and Autism Diagnostic Observation Scale (ADOS) by certified raters to confirm diagnosis. Age of ASD symptom onset was determined by the ADI-R. Regression status was determined based on ADI-R and the shortened Collaborative Programs of Excellence in Autism Regression Interview. Control children were evaluated in the same manner as cases to exclude subjects with any developmental disturbances, including ASD. Questions posed to parents in standardized data collection forms regarding GI symptoms were based on previous work [38]. Symptoms were only reported if the child had experienced the specific GI symptoms, including food allergies and sensitivities, for 3 consecutive months. History of medication use, presence of comorbid conditions, age at first GI episode, and presence and type of food allergies were also acquired through parental questionnaires.

### Tissue lysate preparation

Frozen rectal and cecum biopsies were weighed and then homogenized according to procedures for skeletal muscle as described by Spinazzi et al [39]. Briefly, samples were homogenized using a 0.25 mL dounce homogenizer in 0.04 μl/μg sucrose muscle homogenization buffer with 15 controlled up and down strokes using a Biovortexer 183MC (Biospec Products, Bartelsville, OK, USA). Homogenates were centrifuged at 600 x *g* for 10 min at 4°C, and supernatants were flash frozen in liquid nitrogen and stored at -80°C for no more than 1 week prior to activity assays. Protein concentrations were determined using the BCA protein assay kit (Pierce Inc., Rockford, IL, USA).

### Complex I activity

Electron transport chain (ETC) complex I activity was determined spectrophotometrically by following the oxidation of reduced nicotinamide adenine dinucleotide at 340nm at 30°C in 25μg of lysate according to Saba et al.[40] using a UV-1800 equipped with a temperature-controlled stirring cell (Shimadzu, Kyoto, Japan). The rotenone-sensitive nicotinamide adenine dinucleotide:ubiquinone oxidoreductase activity was calculated from the slopes of the decrease in absorbance of nicotinamide adenine dinucleotide in the sample with and without the addition of rotenone.

### Complex IV activity

ETC Complex IV activity was determined spectrophotometrically by following the oxidation of reduced cytochrome C at 550nm at 30°C in 25μg of lysate according to Saba et al [40]. Reduced cytochrome C was prepared using sodium dithionite as described by Spinazzi et al [39]. The cyanide-sensitive Complex IV activity was calculated from the slopes of the increase in the oxidation of cytochrome C with and without the addition of potassium cyanide.

### Citrate synthase activity

Citrate synthase activity was determined spectrophotometrically by following the conversion of dithionitrobenzoic acid to thionitrobenzoate at 412nm at 30°C in 6 μg of lysate according to Spinazzi et al [39].

### Immunoblotting

Lysates were diluted with sucrose muscle homogenization buffer to 0.5μg/μl. To the lysates, ¼ vol Laemmli sample buffer (BioRad, Hercules, CA, USA) was added with 5% 2-mercaptoethanol and spun at 16,200 x g for 5 minutes. The samples were evenly distributed across three gels such that approximately the same number of samples from each group was contained on each gel to control for any effect of the particular gel run. Supernatants (12μg) were loaded onto Criterion TGX precast gels (Bio-Rad) and run at 150V for 100mins. Proteins were transferred onto a 0.45 μm PVDF membrane (Millipore, Billerica, MA, USA) in CAPS (10mM) with 10% methanol at 150mA for 2hrs. Membranes were incubated with 5% non-fat milk (NFM) in phosphate buffered saline (PBS) overnight at 4°C. Membranes were incubated in 6μg/ml Total OXPHOS Rodent WB antibody cocktail (Abcam, Cambridge, MA, USA) for 2hrs at room temperature. The secondary antibody, anti-mouse Horseradish Peroxidase (Santa Cruz Biotechnology, Dallas, TX, USA), was applied for 2hrs at room temperature at a 1:10000 dilution. Membranes were incubated with Super Signal West Femto (Thermo Scientific, Pittsburgh, PA, USA) for 5mins and proteins were detected using an Image Quant LAS 4000 (GE Healthcare). Signals were analyzed using ImageJ (National Institutes of Health, MD, USA). A non-specific band was used as the loading control instead of a standard housekeeping protein since standard housekeeping protein overlapped in their position on the gel with the ETC complexes. The use of a non-specific band has been used in a wide variety of previous studies [41–45]. The difference in the non-specific band was examined across experimental groups and sampling locations similar to the manner in which the complexes were analyzed and no systematic difference was found across experimental groups or sampling locations. The ratio of the signal derived from the loading control to the enzyme was used to represent protein quantity.

### Statistical analysis

A mixed-model regression [46] was conducted via SAS version 9.3 (Cary, NC, USA) ‘glmmix’ procedure. The mitochondrial ETC complex activity or protein quantity was the response variable with a within-group repeated factor of Area (rectum v cecum) and a between-group factor of Group (ASD v Crohn’s v Control) and ASD vs non-ASD. We present the *overall* difference between the groups (Group Effect), the overall effect of Area (Cecum vs Rectum), the individual effect across groups for each area and the whether the effect of group was different between the two areas (interaction).

For all models, random effects included the intercept and area. F-tests were used to evaluate significance. Planned orthogonal contrasts were used when appropriate. If a group effect was significant, orthogonal contrasts examined the difference between ASD and the other two groups combined; if this comparison was not significant, then the difference between ASD and each other group was examined separately. When an area by group interaction was significant, differences in the cecum and rectum were examined across groups in order to determine whether the cecum or the rectum was driving the difference.

To address the hypothesis that mitochondrial activity would be more atypical in the cecum as compared to the rectum, planned orthogonal contrasts compared the relative difference between the cecum and rectum across groups. A comparison of ASD vs Crohn’s and ASD vs controls were analyzed as well as a difference between ASD vs the combination of Crohn’s and controls (non-ASD).

Initial analysis included participant age at time of collection, sample storage duration and gender but these factors did not show a significant effect and therefore were not included in any final analyses.

## Results

In this study, mitochondria were examined using two approaches. First, the quantity of ETC complex proteins for complexes I-V were examined. Second the activity of two ETC complexes, ETC Complex I and IV, were examined. We examined these parameters of mitochondrial function in both the rectum and the cecum from three clinical populations, children with ASD and those with Crohn’s disease and non-specific GI complaints. We hypothesized that the ASD children would have altered mitochondrial parameters, particularly in the cecum since this is an area of high metabolic activity of the microbiome.

### Electron transport chain complex protein quantity

**Fig 3:**
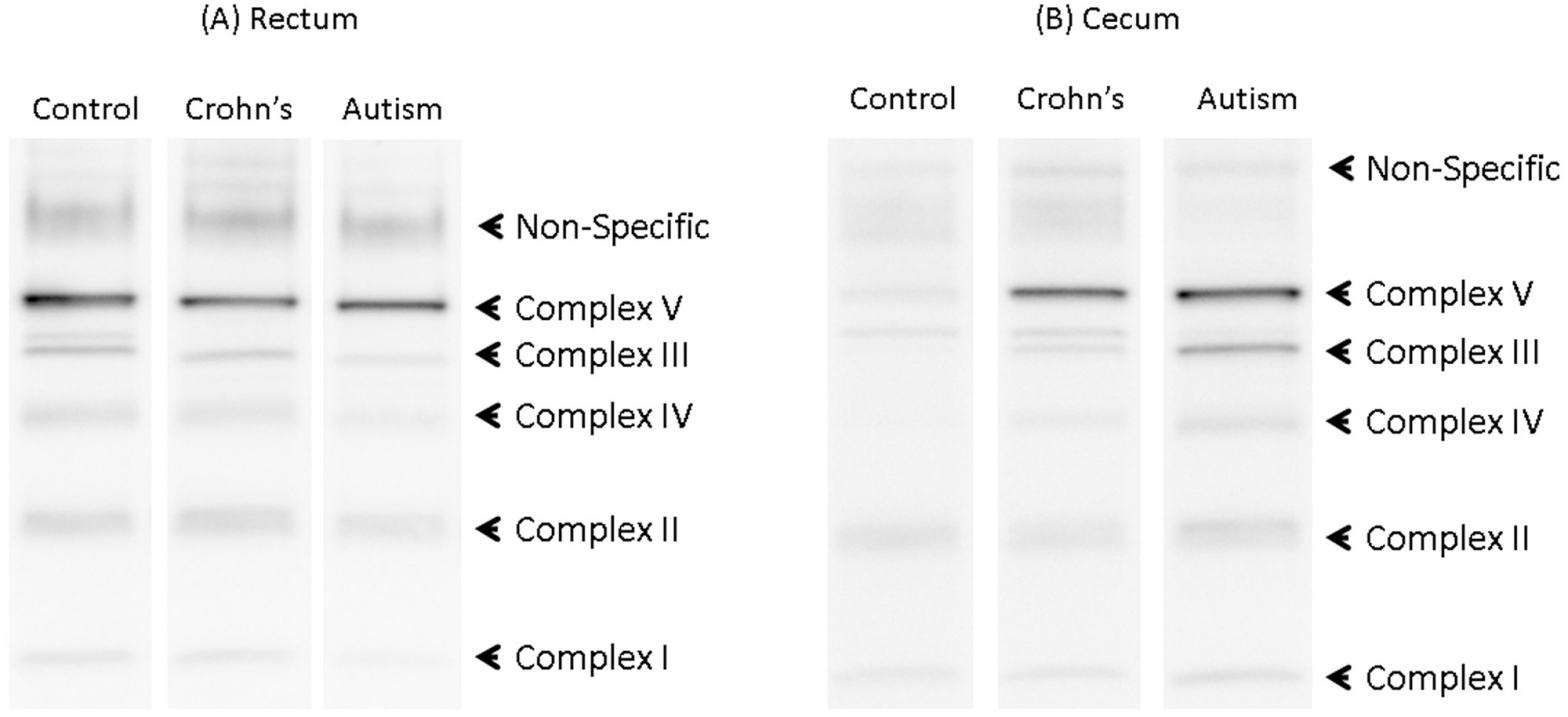
Western Blots of Matched Groups from (A) Rectum and (B) Cecum. Notice that bands for several complexes, particularly complex I, III and IV are darker for the child with autism as compared to controls in the cecum but not the rectum.

Fig 3 shows an example of Western blots for a set of matched rectal and cecum samples.

**Fig 4:**
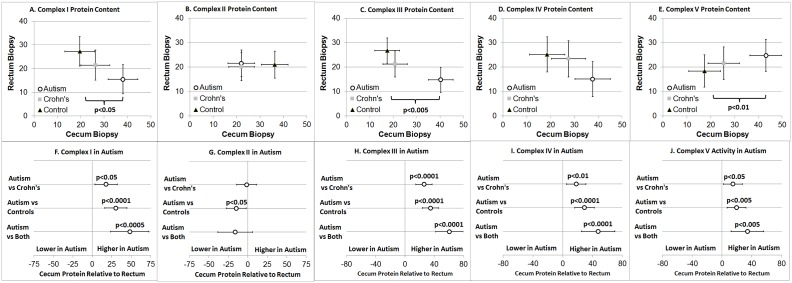
Electron transport chain normalized complex protein quantity. Cecum protein quantity is greater in the autism group as compared to the two control groups for (A) Complex I, (C) Complex III and (E) Complex V. In the cecum, relative to the rectum, protein content is greater in the autism group as compared to the control groups both separately and combined for (F) Complex I, (H) Complex III, (I) Complex IV and (J) Complex V. Error bars represent standard error. The protein quantity values do not have any units because they are normalized. ASD = Autism Spectrum Disorder.

The quantity of complex I protein was significantly higher in the cecum as compared to rectum [F(1,81) = 4.91, p<0.05]. A Group by Area interaction [F(2,81) = 8.78, p<0.0005] was significant (Fig 4A). The quantity of complex I protein was significantly higher in the ASD group as compared to the other groups in the cecum [t(81) = 1.95, p = 0.05] but not in rectum. The quantity of complex I protein was higher in the cecum compared to the rectum when the ASD group was compared to the two other groups separately and in combination (Fig 4F).

The quantity of complex II protein was significantly higher in the cecum as compared to rectum when the data from all groups was considered regardless of group [F(1,81) = 5.01, p<0.05]. The Group by Area interaction [F(2,81) = 3.13, p<0.05] was significant but no specific groups were found to be different when the cecum and rectum were analyzed separately (Fig 4B). However, when the quantity of complex II protein in the cecum relative to the rectum was compared across groups, the ASD group had a lower quantity of complex II protein in the cecum relative to the rectum as compared to the typically developing group but not the Crohn’s Disease group (Fig 4G).

The quantity of complex III protein was significantly higher in the cecum as compared to rectum [F(1,81) = 5.03, p<0.05]. A Group by Area interaction [F(2,81) = 20.49, p<0.0001] was significant (Fig 4C). The quantity of complex III protein was significantly higher in the ASD group as compared to the other groups in the cecum [t(81) = 3.28, p<0.005] but not in rectum. The quantity of complex III protein was higher in the cecum compared to the rectum when the ASD group was compared to the two other groups separately and in combination (Fig 4H).

The quantity of complex IV protein was significantly higher in the cecum as compared to rectum [F(1,81) = 5.88, p<0.05]. A Group by Area interaction [F(2,81) = 9.81, p<0.0005] was significant (Fig 4D). The quantity of complex IV protein was found to be higher in the cecum compared to the rectum when the ASD group was compared to the two other groups separately and in combination (Fig 4I).

The quantity of complex V protein was significantly higher in the cecum as compared to rectum [F(1,81) = 7.19, p<0.01]. A Group by Area interaction [F(2,81) = 4.88, p = 0.01] was significant (Fig 4E). The quantity of complex V protein was significantly higher in the ASD group as compared to the other groups in the cecum [t(81) = 2.66, p<0.01] but not in the rectum. The quantity of complex V protein was higher in the cecum compared to the rectum when the ASD group was compared to the two other groups separately and in combination (Fig 4J).

### Electron transport chain complex activity

**Fig 5:**
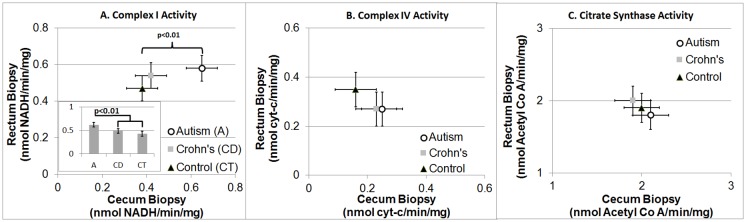
Electron Transport Chain Complex I and IV and Citrate Synthase Activity. Overall complex I activity is greater in the autism group as compared to the two control groups in both the rectum and cecum, when (A) the three groups are analyzed individually. Error bars represent standard error. ASD = Autism Spectrum Disorder.

Overall ETC Complex I activity was significantly different across Groups [F(2,27) = 3.58, p<0.05], due to higher activity in the ASD group as compared to the two other groups [t(27) = 2.55, p<0.05] independent of sampling region (Fig 5A). There was a significantly higher activity of ETC Complex I in the ASD group as compared to the other groups in the Cecum [t(27) = 2.83,p<0.01] but not the rectum. Citrate synthase and ETC Complex IV activity was not significantly different across Areas (Cecum vs Rectum) or Group (Fig 5B and 5C).

## Discussion

In this study, mitochondrial function in the GI tract was tested in the cecum and rectal mucosa and blindly compared between children with ASD to neurotypical children with Crohn’s disease and those with non-specific GI symptoms. Differences in mitochondrial function were found in children with ASD as compared to the other control groups across several ETC complexes suggesting a difference in overall mitochondrial function rather than a change in one specific mitochondrial enzyme. The relative quantity of all ETC complex proteins was higher in the cecum relative to the rectum in children with ASD as compared to the other control children except for ETC Complex II. Examination of ETC complex activity substantiated higher activity in ASD children specific to the cecum for ETC Complex I but not for ETC Complex IV. Citrate synthase activity did not differ across groups suggesting that any changes in ETC activity or protein content was not simply due to mitochondrial proliferation but rather represented up-regulation of multiple ETC complexes. Overall, these findings suggest that the mitochondrial physiology of the GI tract in children with ASD is different than neurotypical children, with some change in mitochondrial function particularly prominent in the cecum. This supports the hypothesis that the pathophysiology of GI abnormalities in children with ASD may be very different than other GI disorders and could be related to changes in mitochondrial function.

Mitochondria are best known for producing energy. Classic mitochondrial disease, which affects about 5% of children with ASD [9], is defined by a deficit in the ability of the mitochondria to make energy and is most typically manifested by a reduction in the activity or protein content of one or more ETC complexes. However, a significant percentage of children with ASD, 30% [9] or more [47, 48], manifest biomarkers of classic mitochondrial disease (e.g., elevated lactate), suggesting that many children with ASD may have a novel type of mitochondrial disorder that is different than classical mitochondrial disease. Indeed, studies have noted a markedly greater than normal activity for ETC Complex 1 in muscle [23] and ETC Complex IV in muscle [49, 50], skin [48] and brain [51] in individuals with ASD. Related to this, a subset of lymphoblastoid cell lines (LCLs) derived from children with ASD have an increase in mitochondrial respiratory activity resulting in a greater sensitivity to oxidative stress [52, 53]. In our recent study of comparing LCLs from males with autistic disorder to their matched typically developing siblings, we found that this atypical elevated mitochondrial respiration is associated with worse repetitive and stereotypical behavior [54].

This study confirms previous reports of increased mitochondrial activity in children with ASD [48–54], specifically increased ETC complex I activity in muscle [23]; and extends this observation to altered ETC complex activity in the GI mucosa. Indeed, in this study, ETC Complex I activity was found to be greater in ASD participants as compared to control participants in GI tissue overall and specifically in the cecum mucosa. This increase in ETC Complex I activity was consistent with the increased protein content in the cecum for ASD participants compared to controls. The increase in Complex III, IV and V protein content suggests that activity of the entire ETC is up-regulated, possibly driven by an increase in NADH (the substrate of Complex I; See Fig 1A). The coordinated increase in multiple ETC complexes suggests that the changes seen are adaptive rather than due to a specific genetic defect which would be most likely to result in a decrease in one specific ETC complex. Still the elevation in Complex I activity in both the cecum and the rectum and the consistency of this findings with previous studies may suggest that the elevation in ETC Complex I activity may have more specificity to ASD than the increases in the other ETC complexes.

The fact that increased ETC complex protein content was primarily seen in the cecum, an area where enteric microbiome fermentation products such as PPA and BUT are abundant, suggests a role for the enteric microbiome in the evolution of mitochondrial abnormalities in children with ASD. A seminal mouse model study demonstrated that a probiotic could significantly attenuate ASD-like behaviors, providing strong evidence for a connection between the microbiome and ASD behaviors [55]. Studies reporting alterations in the enteric microbiome in children with ASD have specifically pointed to an overrepresentation of *Clostridia* spp [26–30] particularly in ASD children with regression [56, 57] and/or those with GI symptoms at or before ASD symptom onset [16]. These overrepresented bacterial are producers of SCFAs such as BUT and PPA which can have significant modulator effects of mitochondrial function.

BUT is major SCFA fermentation product of the same bacteria that have been implicated in ASD, particularly *Clostridia* spp [31]. BUT is a fuel that can be directly integrated into mitochondrial metabolism, and a recent study on germ-free mice suggests that colonocytes may be specifically dependent on BUT for fuel [58]. An increase in ETC Complex I activity without a concomitant increase in Complex II activity could suggest an increase in BUT availability in the lower intestine of children with ASD since BUT enters the citric acid cycle as Acetyl-CoA where it would enhance the production of NADH which would result in an increase in ETC Complex I relative to Complex II (See Fig 1B). Interestingly, BUT also positively modulates neurotransmitter gene expression [59], and rescues ASD type behavior [60] and brain pathology [60, 61] induced by prenatal valproic acid exposure [60] in rodent models of ASD. However, other data suggest that it has a similar effect as PPA on the expression of ASD related genes in cell lines [25] and changes in carnitine and phospholipid metabolism as well as increase in aberrant behavior in animal models [62].

An increase in enzyme activity was not as robust for ETC Complex IV as it was for ETC Complex I. Indeed, ETC Complex IV activity was slightly but not significantly increased in ASD as compared to the non-ASD control groups. We did, however, demonstrate a significantly higher ETC complex IV protein content in the cecum relative to the rectum for ASD as compared to the non-ASD control groups. The fact that this increase was not apparent when the cecum alone was compared across groups, suggests a slightly weaker effect than what was seen for ETC Complex I. This combination of findings may suggest that a greater amount of ETC Complex IV protein is needed to produce the same activity in the cecum, perhaps because ETC Complex IV is functioning inefficiently or because xenobiotic agents are damaging some of the Complex IV proteins. For example, ETC Complex IV is inhibited by inflammation [63].

**Fig 6:**
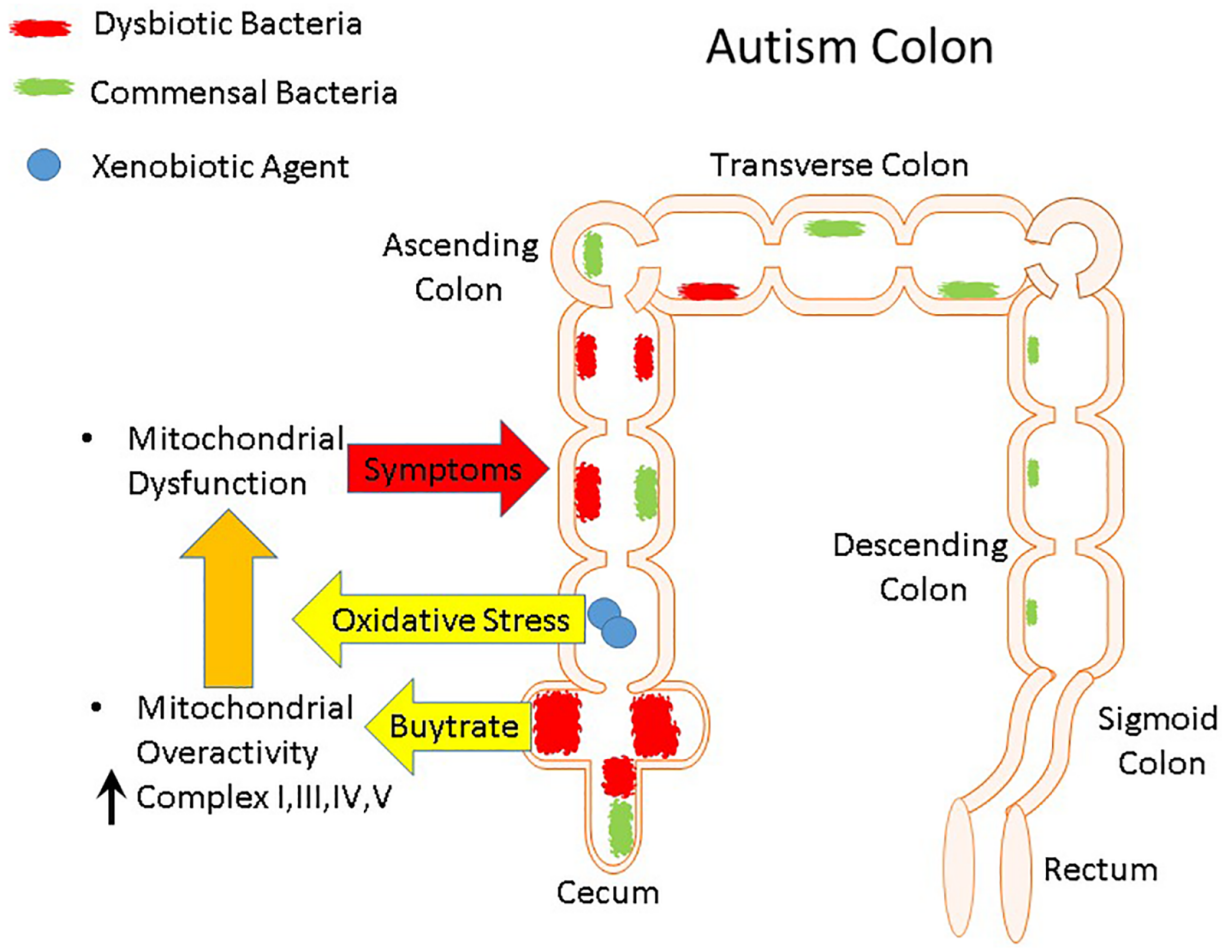
Synthesis of findings of the study. Dysbiotic bacteria in the gastrointestinal tract of individuals with autism produce butyrate that drives the mitochondria to become overactive and very sensitive to oxidative stress. Xenobiotic agents (see previous review [35]) can increase oxidative stress through inflammation or by their intrinsic nature. This results in mitochondrial dysfunction that can contribute to gastrointestinal symptoms such as dysmotility (arrow symbol from mitochondrial dysfunction to colon).

Children with ASD and GI symptoms have also been shown to have higher levels of oxidative stress [64] and higher levels of oxidative stress have been associated with oxidative damage to proteins in ASD [65–69]. Additionally, in LCLs derived from children with ASD, increased respiratory activity is associated with an increased vulnerability to oxidative challenges [52–54], suggesting that the increased oxidative stress associated with ASD could result in dysfunction in overactive mitochondria through the damage of Complex IV. As suggested above, overproduction of BUT could be the driving force behind the overactive mitochondria in GI tissue and xenobiotic agents could be increasing oxidative stress (Fig 6).

Our cell line studies of ASD demonstrated that increased mitochondrial activity is linked to greater vulnerability of the mitochondria to oxidative insults [24, 52, 53, 67]. Interestingly, our data links this increase in mitochondrial respiratory activity to worse repetitive and stereotypical behaviors [54] and such behaviors are indicators for undiagnosed GI abnormalities [13, 14]. As mentioned above, an increase in oxidative stress could result in mitochondrial dysfunction in overactive mitochondria. Such increases in oxidative stress in the GI tract could come from agents such as food additives or other environmental toxicants (Fig 6). Thus, this could explain why some children with ASD are sensitive to particular foods and additives without having classic food allergies and why certain elimination and organic diets may be beneficial [17]. If similar abnormalities in mitochondrial function were occurring in the small bowel where enterocyte function is necessary for the dietary absorption of carnitine and carbohydrates, these abnormalities in enterocyte mitochondrial function could explain decreased carbohydrate transportation and the high prevalence of low serum carnitine levels [20].

Interestingly, PPA may have a paradoxical effect, resulting in increased mitochondrial respiration and an increased sensitivity to physiological stress. In a recent study PPA was found to increase mitochondrial respiration in LCLs in a concentration and exposure time-dependent manner with this increase greater in ASD LCLs as compared to control LCLs [24]. However, when oxidative stress was increased *in vitro*, the same PPA concentration and exposure that increased respiration in LCLs without oxidative stress, caused a significant decrease in respiratory function under conditions of oxidative stress. This reiterates how external stressors can modulate mitochondrial function. This is important since PPA is a SCFA that has several links to ASD [31, 70–74]. For example, PPA induces ASD-like behaviors in adult [23] and juveniles rats [31, 75, 76]. However, in both animal models and humans, the metabolic biomarkers of PAA include an increase in the production of fatty acids, an enhancement of Complex II activity and a reduction of Complex I activity [48], which is opposite of the findings of this study. Indeed, our data suggest that BUT may have a larger role as compared to PPA in individuals with ASD. The importance of BUT in the colon is consistent with a recent study that a deficit in colonocyte bioenergetics is rescued by BUT-producing bacteria [58]. Still, it is important to consider that it may be the relative quantities of these SCFA which can be affected by the relative production from various bacterial populations, the relative breakdown and consumption of SCFAs and the availabilities of specific transporters of SCFAs across the gut wall by enterocytes.

The findings from this study suggest that this novel type of increased mitochondrial activity previously associated with ASD in other tissues may also be found in the GI mucosa, particularly the cecum, and could be related to abnormalities in GI function. The fact that such changes were not also seen in Crohn’s disease, a disease with frank colonic inflammation, suggests that colonic inflammation is not the cause of the changes in mitochondrial function that we measured in the GI tract of children with ASD. The role of mitochondrial function in the GI mucosa and the relationship to GI symptoms observed in children with ASD remains to be determined. However, many of the GI symptoms have been noted in children with mitochondrial disease, such as gastroesophageal reflux, dysmotility, constipation, enterocyte dysfunction, pancreatic dysfunction and vomiting [20].

This and future studies may lead to a better understanding of the pathophysiology associated with GI dysfunction in ASD, leading to new treatment paradigms. Other clinical groups such as those with irritable bowel syndrome might be considered to include in future studies as such individuals, like individuals with ASD, demonstrate alterations in SCFA and psychiatric symptoms [77]. Still the reason for changes in SCFA production in the GI tract should be investigated further. While such changes may be caused simply by an imbalance in the microbiome, it is possible that such SCFA can be induced to heal the GI tract [78]. When considering the study of the microbiome and SCFAs produced it is important to consider dietary intake as changes in prebiotics can alter SCFAs produced. For example inulin-type fructans and arabinoxylan-oligosaccharides increased BUT production in human colon [79]. In addition, many prescription and over the counter medications, some of which are not uncommonly prescribed in ASD (e.g., anti-psychotics), can modulate mitochondrial function [35, 80]. Measurements of SCFAs in the blood, urine or stool could help in the understanding of changes in the microbiome but the reliability and interpretation of such measurements remains to be better validated. Furthermore, other measures of mitochondrial function on the biopsy tissue would help validate our findings but given the limited availability and quantity of GI biopsy tissue in the ASD population, we have attempted to optimize the assays performed.

## Conclusions

This study has demonstrated abnormalities in mitochondrial activity in the lower GI tract of children with ASD, particularly in the cecum. Understanding the connection between mitochondrial dysfunction and GI abnormalities can help clarify the association between behavioral manifestations and GI pathology, the resistance of children with ASD to standard GI treatment and the GI pathology associated with ASD.

## Supporting information

S1 Table Expanded patient characteristics. (DOCX)
