# Supplementary material for: Mitochondrial dysfunction in the gastrointestinal mucosa of children with autism: A blinded case-control study
Source: PLoS One. 2017 Oct 13;12(10):e0186377. doi: 10.1371/journal.pone.0186377 (PMC5640251; doi:10.1371/journal.pone.0186377)
Supplement: S1 Table — (DOCX) [file pone.0186377.s001.docx]

Table S1A. Characteristics for Autism Patients

| **ID** | **Other Medical Conditions** | **Aberrant Behavior** | **Current Language** | **Development** | **Epilepsy** | **Mito Symptoms and Workup** |
| --- | --- | --- | --- | --- | --- | --- |
| 1 | Anemia, SMA syndrome  17yo Male | Aggression,  SBs | Non-verbal | Developmental stagnation at 18 mo | √ | Negative |
| 2 | Hypogammaglobulinemia requiring IVIG, Hypotonia, Food allergies  14yo Male | Aggression,  SIBs, SBs | Non-verbal | Regression in social skills at 44 mo, Gross motor delay, Multiple regressions | √ CPSz | Negative |
| 3 | 2p11.2 ‎‎‎+, Anxiety, Sleep disruption  11yo Male | Aggression  SIBs, SBs | Non-verbal | Regression in speech at 18 mo; Social regression later | √ LKS | MB c/w MD, Brother w/ MD |
| 4 | Food allergies  12yo Female | SIBs | Non-verbal | Regression at 18 mo |  | Negative |
| 5 | Eczema, Food allergy, PANDAS, Advanced bone age  17yo Male | Aggression  OCD, SBs | Non-verbal | Loss of language and social skills at 15 mo, Multiple regressions |  | Mito Labs Abnormal, on cocktail |
| 6 | Cerebral folate deficiency, Sleep disruption, ROM  5yo Female | SBs, Crying | Non-verbal | Loss of language and muscle tone at 8 mo | Profound Atonic | Fatigue |
| 7 | Food allergy, Sleep disruption, SPD2 mutation, Brain cyst / 4^th^ vent shunt  12 year old Female | Hyperactivity, Aggression, SBs | Non-verbal | Slow regression in language and social skills starting at 18 mo | Abnormal EEG | Complex I Deficiency |
| 8 | Severe food allergy, Macrocephaly  17 year old Male | SIBs | Non-verbal | Loss of language and social skills at 18 mo |  | Negative |
| 9 | Environmental allergies  7 year old Male | SBs, Crying | Limited | Loss of language and social skills at 24 mo |  | Negative |
| 10 | Cyclic vomiting, Hypotonia,  15 year old Male | Aggression | Non-verbal | Loss of language and social skills at 15 mo | √ LKS | Negative |

Abbreviations: CPSz = Complex Partial Seizures; EEG = Electroencephalogram; GERD = Gastroesophageal Reflux Disease; LKS = Landau-Kleffner syndrome; IVIG = Intravenous Immunoglobulin; MB = Muscle Biopsy; MD = Mitochondrial Disease; mito = mitochondrial; mo = months old; ND = Not Done; ROM = Recurrent otitis media; SBs = Repetitive Stereotypic Behavior; SIBs = Self-Injurious Behaviors; SMA = Superior mesenteric artery; OCD = Obsessive-Compulsive Disorder; PANDAS = Pediatric Autoimmune Neuropsychiatric Disorders Associated with Streptococcal Infections; yo = year old

Table S1B. Characteristics of Control Groups

| ID | **Medical Conditions and Significant Medications** | **Mitochondrial Symptoms** |  |
| --- | --- | --- | --- |
| **Neurotypical with Non-Specific GI symptoms** | | | |
| 1: | 7yo Male | No Symptoms |  |
| 2: | 11yo Female with BREC | No Symptoms |  |
| 3: | 11yo Male | No Symptoms |  |
| 4: | 18 yo Male | No Symptoms |  |
| 5: | 13 yo Male with Hashimoto’s thyroiditis | No Symptoms |  |
| 6: | 17 yo Male | No Symptoms |  |
| 7: | 6yo Female | No Symptoms |  |
| 8: | 17 yo Male | No Symptoms |  |
| 9: | 13 yo Female | No Symptoms |  |
| 10. | 15 yo Male with High-functioning Autism^a^ |  |  |
| **Neurotypical Patients with Crohn’s Disease** | | | |
| 1 | 6 yo Male | No Symptoms |  |
| 2: | 12yo Female treated with Immunosuppression and Prednisone | Fatigue |  |
| 3: | 16 yo Male | Fatigue |  |
| 4: | 14 yo Male treated with infliximab | No Symptoms |  |
| 5: | 7 yo Female | No Symptoms |  |
| 6: | 11 yo Female treated with infliximab | No Symptoms |  |
| 7: | 17 yo Male treated with mesalamine | No Symptoms |  |
| 8: | 16yo Male | No Symptoms |  |
| 9: | 15yo Male underweight with poor med compliance | No Symptoms |  |
| 10: | 11 yo Male treated with Infliximab, mesalamine, metronidazole | No Symptoms |  |

a This high-functioning child with autism was misclassified as typically developing during the blind analysis. Thus, in all analyzes this individual was included in the typically developing group.

Abbreviations: yo = year old; BREC = benign rolandic epilepsy of childhood; RLQ = Right Lower Quadrant
